# Cerebral infarction pattern in tuberculous meningitis

**DOI:** 10.1038/srep38802

**Published:** 2016-12-13

**Authors:** Mei-Ling Sharon Tai, Shanthi Viswanathan, Kartini Rahmat, Hazman Mohd Nor, Khairul Azmi Abdul Kadir, Khean Jin Goh, Norlisah Ramli, Fatimah Kamila Abu Bakar, Norzaini Rose Mohd Zain, Jun Fai Yap, Beng Hooi Ong, Mohd Hanip Rafia, Chong Tin Tan

**Affiliations:** 1 Division of Neurology, Department of Medicine, University Malaya, Lembah Pantai, 50603 Kuala Lumpur, Malaysia; 2 Department of Neurology, Hospital Kuala Lumpur, Jalan Pahang, 50586 Kuala Lumpur, Malaysia; 3 Department of Biomedical Imaging, Faculty of Medicine, University Malaya, Lembah Pantai, 50603 Kuala Lumpur, Malaysia; 4 Department of Radiology, Hospital Kuala Lumpur, Jalan Pahang, 50586 Kuala Lumpur, Malaysia; 5 Department of Medicine, Hospital Alor Setar. Jalan Langgar, 05460 Alor Setar, Malaysia.

## Abstract

Tuberculous meningitis (TBM) causes significant morbidity and mortality. The primary objective was to re-examine the concept of “TB zone” and “ischaemic zone” in cerebral infarction in patients with tuberculous meningitis. The secondary objective was to evaluate cerebral infarction, vasculitis and vasospasm in tuberculous meningitis infections. Between 2009 and 2014, TBM patients were recruited. Neuroimaging was performed and findings of cerebral infarction, vasculitis and vasospasm were recorded. Infarcts were classified based on arterial supply and Hsieh’s classification. Fifty-one TBM patients were recruited of whom 34 patients (67%) had cerebral infarction. Based on Hsieh’s classification, 20 patients (59%) had infarcts in both “TB zone” and “ischaemic zones”. 12 patients (35%) had infarcts in “ischaemic zone” and two (6%) patients had infarcts in “TB zone”. In terms of vascular supply, almost all patients (35/36) had infarcts involving perforators and cortical branches. 25 patients (73%) and 14 patients (41%) had infarcts supplied by lateral lenticulostriate and medial lenticulostriate arteries respectively. 15 patients (37%) had vasculitis. Vasospasm was present in six patients (15%). 29 patients (85%) with cerebral infarction also had leptomeningeal enhancement (p = 0.002). In summary, infarcts involved mainly perforators and cortical branches, rather than “TB zone” versus “ischaemic zone”.

As we entered the second decade of the twenty first century, tuberculosis (TB) remained an infection of global importance. In 2012, there were 8.6 million new and 1.3 million deaths due to tuberculosis^1^. Tuberculous meningitis (TBM) occurs in 4% of patients with tuberculosis^2^. Tuberculous meningitis causes significant morbidity and mortality, especially if diagnosis and treatment are late^1^.

Cerebral infarction occurs in 15–57% of tuberculous meningitis patients, mainly during stage three of the illness^3^. According to a study published by Hsieh and colleagues, 75% of infarcts occurred in the “TB zone” supplied by the medial lenticulostriate and thalamoperforating arteries, whereas only 11% occurred in the “ischaemic zone” supplied by the lateral lenticulostriate, anterior choroidal and thalamogeniculate arteries^4^.

This concept has been widely accepted. For example, it was discussed in a review on stroke in tuberculous meningitis in 2011^3^, and also in a chapter on tuberculous meningitis of an important neurology textbook on tuberculous meningitis in the developing world^5^.

The primary objective of the present study was to re-examine the concept of “TB zone” and “ischaemic zone” in cerebral infarction in patients with tuberculous meningitis. The secondary objective was to evaluate cerebral infarction, vasculitis and vasospasm in tuberculous meningitis infections.

## Methodology

This study was a transversal descriptive study on the patients with tuberculous meningitis.

### Patients

Between 2009 and 2014, we collected data from all patients with tuberculous meningitis at University Malaya Medical Centre (UMMC) and Kuala Lumpur General Hospital, two large tertiary referral hospitals in Kuala Lumpur, Malaysia.

Patients admitted with tuberculous meningitis to the neurology wards of University Malaya Medical Centre and Kuala Lumpur General Hospital were included in the study. Tuberculous meningitis patients who did not undergo Computed Tomography scan of brain within one month of hospitalization were excluded from the study.

Tuberculous meningitis was categorised as “definite”, “probable” and “possible”. This classification was based on a modification of the Thwaites criteria^6^. Tuberculous meningitis was defined as “definite” when cerebrospinal fluid (CSF) acid-fast bacilli direct (AFB) smear, mycobacterial culture, polymerase chain reaction (PCR) or histopathological findings were positive. Tuberculous meningitis was defined as “probable” if the patients had one or more of the features of active pulmonary tuberculosis, acid-fast bacilli in specimens other than cerebrospinal fluid or extrapulmonary tuberculosis^6^.

Tuberculous meningitis was termed as “possible” if patients had at least four of the following features: cerebrospinal fluid pleocytosis, lymphocyte predominance in the cerebrospinal fluid, cerebrospinal fluid glucose to plasma glucose ratio of less than 0.5, turbid cerebrospinal fluid, a period of illness lasting for more than five days, absence of Cryptococcal infection, altered conscious level, focal neurological signs or response to antituberculous drugs^6^,^7^.

The study was approved by the Institutional Review Board of University Malaya Medical Centre and the Ministry of Health, Malaysia. All patients or their legally acceptable representatives provided written informed consent for participation. The methods were carried out *in accordance with* the approved guidelines. The ethical criteria for the study were fulfilled. The privacy of the information has been maintained. We collected data on demographic and clinical characteristics such as age, gender, ethnic group, disease duration, as well as symptoms and signs on admission.

Information from tests of cerebrospinal fluid on admission was recorded. Information was collected on cerebrospinal fluid opening pressure, cerebrospinal fluid glucose, protein, white cell count (lymphocyte and neutrophil differential count), tuberculous polymerase chain reaction, acid-fast bacilli smear, mycobacterial culture and sensitivity to antituberculous medications. In addition, the presence of pulmonary or tuberculous disease of spine (TB spine), was documented. The presence of other predisposing illnesses such as human immunodeficiency viral illness (HIV) was recorded.

The severity of meningitis at the time of admission was recorded according to British Medical Council criteria^6^. Patients in stage 1 had Glasgow coma scale (GCS) of 15/15 with meningeal signs but no focal neurological deficits. Patients in stage 2 had altered mentation or focal neurological deficit (GCS 11–14). Patients in stage 3 had either stupor, delirium, hemiplegia or paraplegia (GCS ≤ 10)^6^. Advanced stages were defined as stages 2 and 3. The patients’ clinical course was assessed regularly.

All the patients received treatment with first line antituberculous therapy consisting of an intensive phase of four drugs (ethambutol, isoniazid, rifampicin and pyrazinamide) followed by a maintenance phase of two drugs (isoniazid, rifampicin). The total duration of antituberculous therapy was 12–18 months based on the British Infection Society guidelines^8^. All patients had supervised antituberculous therapy during hospitalization and during follow-up as outpatients’^9^. Corticosteroid treatment was administered to patients who had severe tuberculous meningitis.

Modified Rankin scale (MRS) was used to evaluate clinical outcome at three months. The MRS ranged from 0 to 6^6^,^10^. Scores of 0–2 were defined as good outcome, while scores of 3–6 were defined as poor outcome^6^,^10^.

### Methods

Computed Tomography scan of the brain was performed on admission and the data was documented. Magnetic resonance imaging (MRI) of brain was performed using a 3.0-Tesla Signa HDx MR system (GE healthcare). The sequences included T1 weighted (T1W) image without and with gadolinium contrast enhancement, T2 weighted (T2W) image, T2 fluid-attenuated inversion recovery (T2 FLAIR) image, diffusion-weighted image (DWI), apparent diffusion coefficient (ADC) and magnetic resonance angiography (MRA) sequences. Serial Computed Tomography of brain and Magnetic Resonance Imaging of brain studies were performed.

All the scans were reviewed by neuro-radiologists (HMN and KAAK) and neurologists (MLST and CTT). The neuro-radiologists who reviewed the scans (HMN and KAAK), were blinded to the patients’ condition and disease stage. All abnormalities were recorded. In particular, the patients were assessed for leptomeningeal enhancement, cerebral infarction, hydrocephalus, tuberculoma and vascular pathology such as vasculitis and vasospasm.

The tuberculous meningitis patients were assessed for cerebral infarction by two different methods. The first classification was based on vascular supply. The various arteries were medial lenticulostriate arteries, lateral lenticulostriate arteries, cortical branches, terminal penetrating arteries of the basilar artery and perforators of the posterior cerebral artery (PCA).

The A1 segment of the anterior cerebral artery (ACA) or the deep branches of the anterior cerebral artery give rise to the medial lenticulostriate arteries. Medial lenticulostriate arteries supply the head of the caudate nucleus, the genu and anterior limb of the internal capsule and the anterior part of globus pallidus^11^,^12^.

The lateral lenticulostriate arteries are branches of the horizontal M1-segment of the middle cerebral artery. These are deep penetrating branches or lenticulostriate branches of middle cerebral artery^11^,^12^. The arteries supply the head and body of caudate nucleus, most of globus pallidus (outer), putamen, posterior limb of internal capsule, corona radiata and external capsule^11^,^12^.

Posterior thalamoperforating arteries are branches from the P1 segment of posterior cerebral artery (PCA) to supply the midbrain and thalamus^11^,^12^. The thalamogeniculate artery originates from the ambient or P2 segment of the posterior cerebral artery^9^.

The second classification was based on Hsieh *et al*.’s “TB zone” and “ischemic zone”^4^. The “TB zone” is the area supplied by the medial lenticulostriate, thalamotuberal and thalamoperforating arteries. The “TB zone” consists of head of the caudate nucleus, genu and anterior limb of internal capsule and anteromedial thalamus^4^. The “ischaemic zone” is supplied by lateral lenticulostriate, anterior choroidal and thalamogeniculate artery^4^. The “ischemic zone” consists of the lentiform nucleus, posterolateral thalamus and posterior limb of internal capsule^4^. The two methods of infarct classification were compared.

An assessment of vasculitis and vasospasm was also performed. Vasculitis was defined as the presence of localized and short segment stenosis/narrowing or beading on computed tomography angiography (CTA) or magnetic resonance angiography (MRA). Stenosis was assessed by comparing the diameter of the affected segment of the vessel with the diameter of the nearest normal vessel segment. Beading was defined as alternating, short, regularly and spaced segments of stenosis with short normal or dilated intervening segments. Vasospasm was defined as long segment reversible narrowing on serial computed tomography angiography or magnetic resonance angiography of brain.

The patients were monitored at regular intervals by clinical, cerebrospinal fluid parameters and neuroimaging findings. Lumbar puncture was performed at regular intervals. Computed tomography/Magnetic resonance imaging of brain was repeated 1–2 months after admission and when patients showed clinical deterioration.

### Statistical analysis

All descriptive statistics were done with Statistical Package for Social Sciences, SPSS (Version 18.0, SPSS Inc., Chicago, USA). Continuous variables were expressed as means and standard deviation. In addition, categorical variables were expressed as frequencies and percentages. Chi square test (or Fisher exact test) was performed to analyse the association of cerebral infarcts with vasculitis, vasospasm, leptomeningeal enhancement and outcome.

## Results

### Demography characteristics of TBM patients

Out of the 53 tuberculous meningitis patients seen between 2009 and 2014, 51 patients (96.2%) were recruited and included in the analysis (Table 1). Two patients who did not undergo computed tomography scan of brain within the first month of hospitalisation were excluded.

### Neuroimaging results of tuberculous meningitis patients

**Figure 1:**
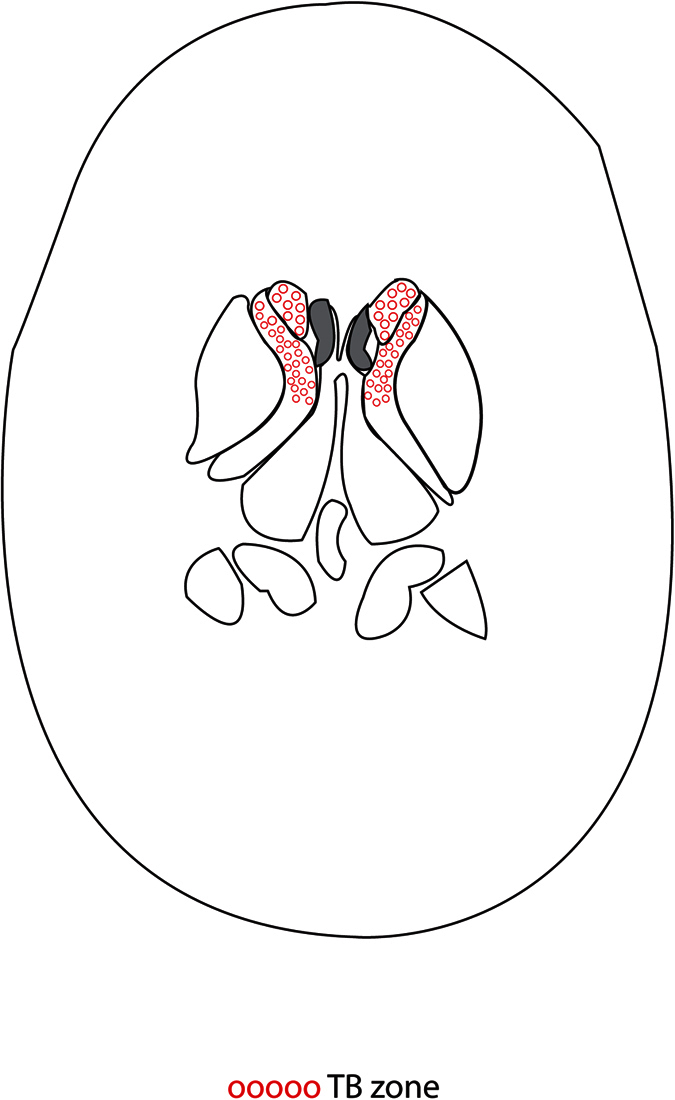
Diagram of “TB zone”.

**Figure 2:**
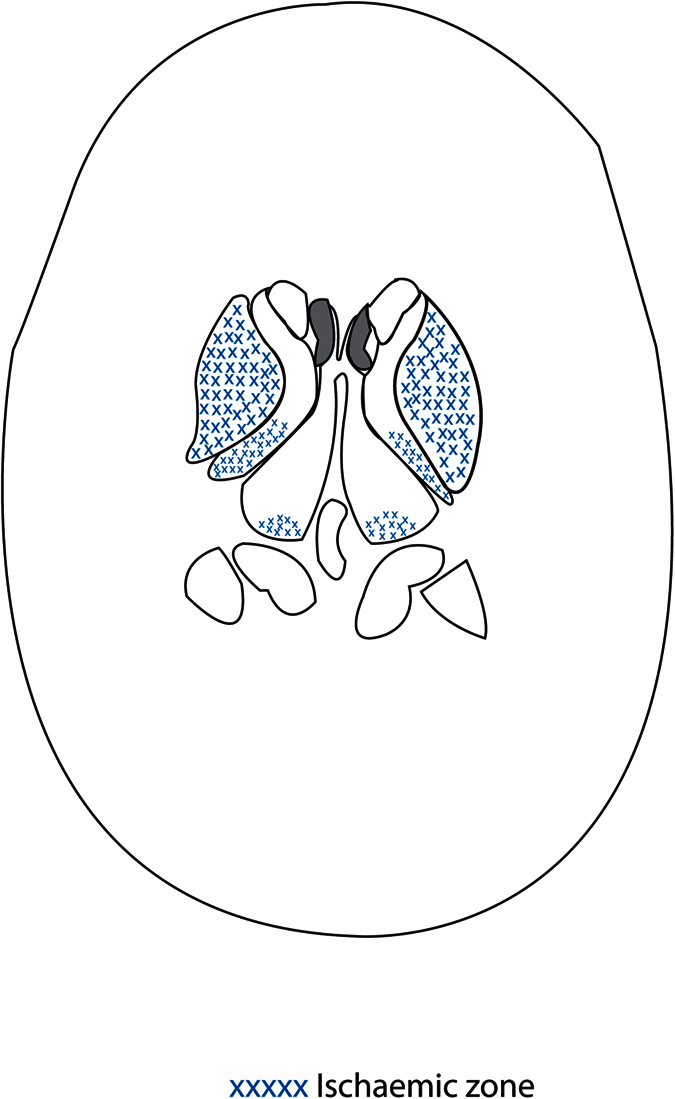
Diagram of “Ischaemic zone”.

When the infarcts in tuberculous meningitis patients were categorized according to Hsieh’s classification, twenty patients (59%) had infarcts in both the “TB zone” and the “ischaemic zone”. Figure 1 shows the diagram of “TB zone” and Fig. 2 shows the diagram of “ischaemic zone”. Twelve patients (35%) had infarcts in the “ischemic zone” only and two (6%) patients had infarcts in the “TB zone” only (Table 2).

When cerebral infarction was classified according to vascular supply, 25 patients (73%) had infarcts in territory supplied by lateral lenticulostriate arteries, while 14 patients (41%) had infarcts in the territory supplied by medial lenticulostriate arteries. 13 patients (38%) had infarcts in the area supplied by perforators of the posterior cerebral artery. Ten patients (29%) developed infarcts in areas supplied by cortical branches.

Thirty-three patients (97%) had infarcts associated with pathology of both the perforators and cortical branches. One patient (3%) had an infarct in a region supplied by cortical branch only.

No patient had infarct due to total occlusion of major intracranial arteries. Moreover, no patient had infarct in the internal borderzone of middle cerebral artery which was typical for patients with atherosclerotic disease.

Infarction of cortical areas was less common (18%). Enhancement was seen in cortical infarcts but not basal ganglia and thalamic infarcts.

Of the forty patients who underwent either computed tomography angiography, magnetic resonance angiography or both tests, 15 patients (37%) had vasculitis, which was most common in the middle cerebral artery. Vasospasm was present in six patients (15%), commonly found in the middle cerebral artery.

**Figure 3:**
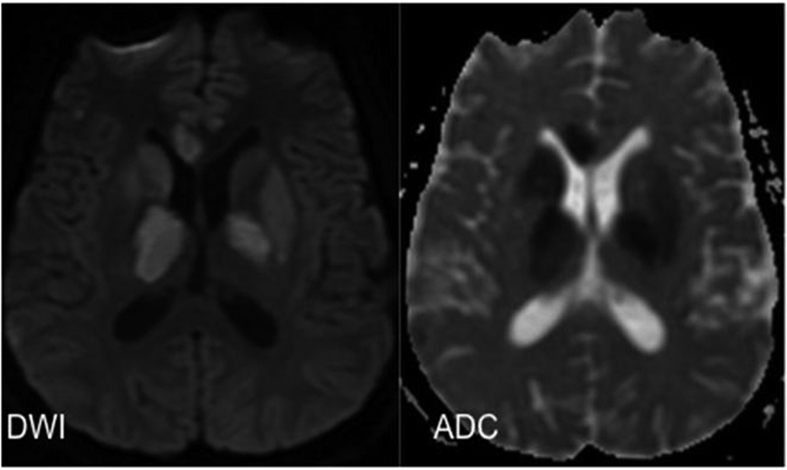
MRI brain ADC/DWI show restricted diffusion in the right caudate nucleus, both thalami, both lentiform nuclei and genu of right corpus callosum, consistent with acute infarction.

**Figure 4:**
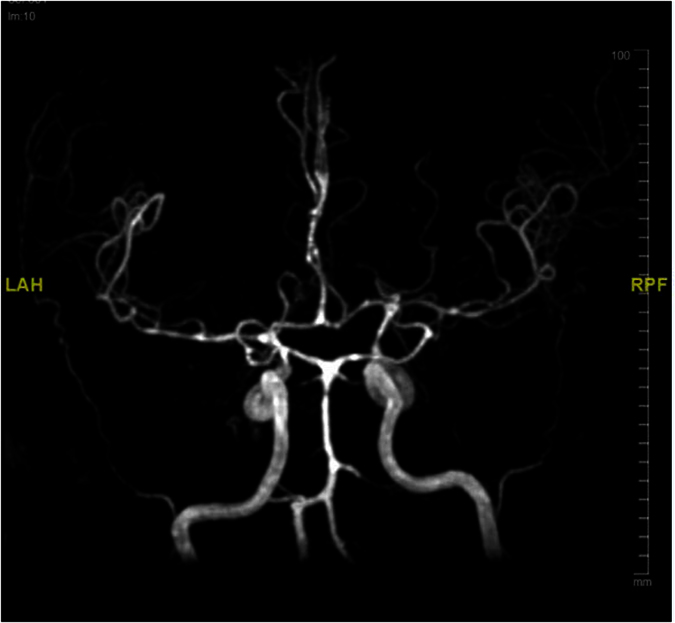
MRA Circles of Willis demonstrates vasospasm involving the basilar artery, bilateral MCA, bilateral ACA and bilateral distal ICA.

**Figure 5:**
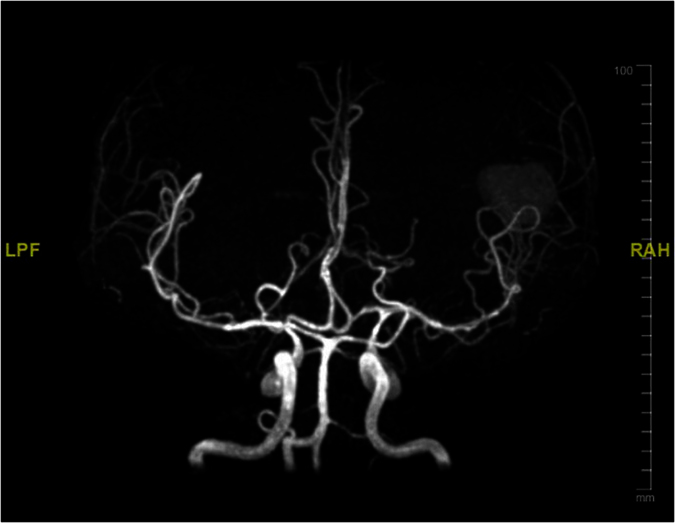
Repeat MRA one month later shows improvement of the narrowing.

Table 3 shows association of cerebral infarcts with vasculitis, vasospasm, leptomeningeal enhancement and outcome. Cerebral infarction was associated with leptomeningeal enhancement with statistical significance (p = 0.002). Figure 3 shows magnetic resonance imaging of the brain images (diffusion weighted image, apparent diffusion coefficient) of a tuberculous meningitis patient with acute infarction in the basal ganglia and thalamus. Figure 4 shows the vasospasm of the cerebral vessels, and Fig. 5 shows the improvement of the vasospasm.

There was no statistical significance when comparing vasculitis and vasospasm between good outcome and poor outcome groups (p = 1.00).

## Discussion

Our study showed that cerebral infarction was seen in 67% of our tuberculous meningitis patients in this study. There were more cases of infarcts in patients in this study compared to previously published reports (6–47%)^13^.

In the present study, the most common site of cerebral infarction was in the basal ganglia, found in 49% of our patients, and this was comparable to previous reports of 24–59% of tuberculous meningitis patients^3^,^14^,^15^,^16^,^17^,^18^,^19^,^20^.

The study by Hsieh *et al*.^4^. showed that infarcts were more common in the “TB zone”^4^. However, the areas of infarct in our study did not correspond to their findings. Overall, there were 12 patients (35% of all patients with infarcts) with infarcts at the “ischaemic zone”, compared to only two patients (6%) where the infarct was confined to the “TB zone”.

In contrast, involvement of perforators and the terminal cortical branches was demonstrated in 33 of 34 patients with infarcts. One patient had infarct involving the terminal cortical branch only. These infarcts mainly involved the lateral lenticulostriate arteries (73%), medial lenticulostriate arteries (41%), and perforators from posterior cerebral artery (38%).

Of note, infarcts were adjacent to basal ganglia infarcts in each of the three patients with corona radiata infarcts. This can be explained by perforator artery disease rather than middle cerebral artery main trunk disease.

Infarcts from total occlusion of major intracranial arteries was not seen among our patients, unlike infarcts due to atherosclerosis. The predominant involvement of the perforators and terminal cortical branches rather than occlusion of the major intracranial arteries, thus represented other characteristic neuroimaging features characteristic of tuberculous meningitis, distinct from infarcts in atherosclerosis.

Forty-one percent of patients with infarcts had bilateral symmetrical infarcts involving the same corresponding territory and forty-one percent of patients with infarcts had infarcts involving multiple vascular territories. This also represents a difference compared to infarcts seen in atherosclerosis^4^,^21^. Some of our patients had infarcts involving same anatomical sites at both sides: 38% with thalamic infarcts and 31% with caudate infarcts. Infarcts involving multiple vascular territories and bilateral infarcts also represented imaging features characteristic of tuberculous meningitis.

Close to two-fifths of tuberculous meningitis patients in the current study had vasculitis. The predominant involvement of the large intracranial vessels was consistent with previous studies^3^,^18^,^19^. This may be explained by the common location of the meningitis at the base of brain and the adjacent Sylvian fissures close to these arteries. Vasculitis in basilar artery and vertebral artery as seen in our patients, has not been previously described in the literature to our knowledge.

Studies on vasospasm in tuberculous meningitis have been rare^3^. In the present study, the most commonly affected vessels in vasospasm were the middle cerebral artery, followed by terminal internal carotid artery, basilar, posterior cerebral artery and vertebral artery.

Cerebral infarction is associated with leptomeningeal enhancement in TBM. The thickness of leptomeningeal enhancement in brain scan predicts amount of exudates in the brain. The exudate at the basal region surrounds the arteries, leading to arterial narrowing and subsequently stroke^14^,^20^. The intense inflammation also causes vasculitis and vasospasm in the nearby vessels^14^,^20^.

Cerebral infarction in TBM involved especially the perforators and terminal cortical branches, rather than “TB zone” versus “ischaemic zone”. We believe that the vascular supply classification is more accurate than the classification of “TB zone” vs “ischaemic zone”. Proper recognition of cerebral infarction in TBM using the framework of vascular supply facilitates early diagnosis of TBM by neuroimaging.

Compared to the other tests, we have found brain imaging to be helpful in the early diagnosis of TBM. Neuroimaging of brain can be done rapidly. By comparison, CSF TB PCR result takes several days before it is known to the clinician. In addition, only 29% of our patients have positive CSF for TB PCR. Moreover, there is a long delay before CSF mycobacterium tuberculosis culture result is available.

By proper and early identification of cerebral infarcts in TBM infections using the vascular supply classification, adequate treatment can be given early as well. Aspirin can be administered to the TBM patients with vasculitis^3^,^22^. In addition, adjunctive corticosteroid is beneficial in reducing morbidity and mortality in advanced TBM^3^,^22^. There are also potential therapies for vasospasm^23^. Therefore, we propose that the vascular supply classification in cerebral infarcts due to TBM be implemented in the clinical setting. We believe that this will help improve clinical outcome in TBM patients in the clinical setting even further.

In addition, through early diagnosis with vascular supply framework and treatment of TBM, other severe and potentially life-threatening complications, such as, hydrocephalus can be prevented or minimised. Furthermore, serial brain scans can be done to monitor for worsening or new infarcts, vasculitis, vasospasm and leptomeningeal enhancement.

Our study has several limitations. This study is a descriptive study, and the findings cannot be extrapolated in other populations. Moreover, only descriptive statistics and chi-square analysis were performed. Another limitation was the absence of control group. A case-control study may be useful.

In conclusion, this study showed that cerebral infarction in tuberculous meningitis involved mainly the perforators and terminal cortical branches, rather than “TB zone” versus “ischaemic zone”. The vascular supply classification is an accurate method of classifying cerebral infarcts in TBM. Proper recognition of cerebral infarction in TBM using the framework of vascular supply facilitates early diagnosis and management of complications of TBM.

## Additional Information

**How to cite this article:** Tai, M.-L. S. *et al*. Cerebral infarction pattern in tuberculous meningitis. *Sci. Rep.* **6**, 38802; doi: 10.1038/srep38802 (2016).

**Publisher's note:** Springer Nature remains neutral with regard to jurisdictional claims in published maps and institutional affiliations.

## Figures and Tables

**Table 1:** Baseline characteristics, cerebrospinal (CSF) and sputum results of TB meningitis patients.

	Patients, n = 51
Age (mean ± SD)	35.1 ± 12.9
Gender (n, %)	
Male	30 (59%)
Female	21 (41%)
Ethnic group (n, %)	
Malay	18 (35%)
Chinese	10 (20%)
Indian	12 (23%)
Non-Malaysians	11 (22%)
Clinical features (n, %)	
Fever	39 (76%)
Headache	34 (67%)
Altered sensorium/	33 (65%)
Confusion	
Vomiting	27 (53%)
Loss of appetite	25 (49%)
Loss of weight	17 (33%)
Hemiparesis/paraparesis	15 (29%)
Neck stiffness	14 (27%)
Stage of illness on admission (n, %)	
Stage 1	14(27%)
Stage 2	26(51%)
Stage 3	11(22%)
Other TB sites (n, %)	
Pulmonary tuberculosis	20 (39%)
Pleural tuberculosis	4 (8%)
Tuberculosis disease of spine	9 (18%)
Other medical illnesses (n, %)	
Human immunodeficiency viral illness	7 (14%)
Diabetes mellitus (DM)	5 (10%)
Hypertension (HT)	4 (8%)
Others	8(16%)
Cerebrospinal fluid results on admisssion	
Opening Pressure, cm H_2_O (mean ± SD)	25.50 ± 15.86(range 1.5–75)
White blood cells, cells/ml (mean ± SD)	169.49 ± 241.58 (range 0–1152)
Lymphocyte differential in percentage (mean ± SD)	55.97 ± 39.25 (range 0–100)
Neutrophil differential in percentage (mean ± SD)	39.91 ± 39.95 (range 0–100)
Glucose, mmol/L (mean ± SD)	1.89 ± 1.31 (range 0.3–7.2)
Protein, g/L (mean ± SD)	3.23 ± 4.80 (range 0.19–21.96)
Acid Fast-bacilli direct smear (n, %)	3(6%)
Mycobacterial culture positive (n, %)	25 (49%)
Tuberculous Polymerase chain reaction (n, %)	
-positive (out of 34 samples)	10 (29%)
Sputum (n, %)	
Mycobacterial culture positive	14(27%)
Diagnosis (n, %)	
Definite	32(63%)
Probable	8(16%)
Possible	11(21%)
Outcome/Modified Rankin Scale (MRS) at 3 months (n, %)	
0	5(10%)
1	9(18%)
2	1(2%)
3	8(15%)
4	6(12%)
5	5(10%)
6	17(33%)

**Table 2:** Neuroimaging results of tuberculous meningitis patients.

	Patients, n = 51	Left (n, %)	Right (n, %)	Both (n, %)
	(n, %)			
**Cerebral infarcts** (n, % out of all TBM patients)	34(67%)			
Thalamus	13 (26%)	5(10)	3(6)	5(10)
-Anteromedial thalamus	12 (24%)			
-Posterolateral thalamus	1(2%)			
**Basal ganglia**	25(49%)			
Globus pallidus	14 (28%)	4(8)	9(18)	1(2)
Putamen	12(24%)	6(12)	4(8)	2(4)
Caudate	13 (26%)	4(8)	5(10)	4(8)
-Head of caudate	12 (24%)			
-Head and body of caudate	1(2%)	1(2)		2(4)
Temporal	6(12%)	3(6)		
-Temporal infarct with leptomeningeal enhancement	3(6%)			
Parietal	3(6%)	1(2)	2(4)	0
- Parietal infarct with leptomeningeal enhancement	3(6%)			
Frontal	2(4%)	2(4)	0	0
- Frontal infarct with leptomeningeal enhancement	0			
Occipital	1(2%)	0	0	1(2)
- Occipital infarct with leptomeningeal enhancement	0			
Corona radiata	3(6%)	3(6)	0	0
- Corona radiata infarct adjacent to the basal ganglia infarct	3(6%)			
Corpus callosum	3(6%)	1(2)	1(2)	1(2)
Internal capsule	5(10%)	5(10)	0	0
-Anterior limb	1(2%)			
-Genu	2(4%)			
-Posterior limb	3(6%)			
Insula	2(4%)			
Midbrain	3(6%)			
Pons	5(10%)			
External capsule	5(10%)			
Cerebellar vermis	1(2%)			
Cerebellar folia	1(2%)			
Middle cerebellar peduncle	2(4%)			
Hypothalamus	1(2%)			
Bilateral symmetrical infarcts	14 (27% of all TBM patients, 41% of patients with infarcts)			
Multiple infarcts	14 (27% of all TBM patients, 41% of patients with infarcts)			
**Classification of cerebral infarction according to vascular supply**				
(n, % out of all 34 patients with infarcts)				
Medial lenticulostriate arteries (terminal perforator of anterior cerebral artery)	14(41%)			
Lateral lenticulostriate arteries	25(73%)			
Perforators from posterior cerebral artery	13(38%)			
Cortical branches	10(29%)			
Terminal penetrating arteries from basilar artery	4(12%)			
Superior cerebellar artery	1(3%)			
Anterior inferior cerebellar artery	2(6%)			
**Classification of cerebral infarction according to Hsieh’s classification**^7^				
(n, % out of all 34 patients with infarcts)				
TB zone	2(6%)			
Ischaemic zone	12(35%)			
Combined TB zone and ischaemic zone	20(59%)			
**Vasculitis** (n, %)	15 (37%)			
Terminal internal carotid artery	4(10%)	1(2.5)	3(7.5)	0
Middle cerebral artery	10(25%)	5(12.5)	2(5)	3(7.5)
Anterior cerebral artery	5(12%)	1(2.5)	2(5)	2(5)
Posterior cerebral artery	4(10%)	1(2.5)	0	3(7.5)
Basilar artery	3(7%)			
**Vasospasm** (n, %)	6(15%)			
Terminal internal carotid artery	4(10%)	0	2(5)	2(5)
Middle cerebral artery	6(15%)	1(2.5)	3(7.5)	2(5)
Anterior cerebral artery	1(2%)			1(2.5)
Posterior cerebral artery	2(5%)	1(2.5)	0	1(2.5)
Basilar artery	2(5%)	0	0	1(2.5)
Vertebral artery	1(2%)			
**Leptomeningeal enhancement** (n, %)	36(71%)			
Interpeduncular fossa	28(55%)			
Prepontine fissure	28(55%)			
Quadrigeminal cistern	15(29%)			
Ambient cistern	13(25%)	0		
Sylvian fissure	22(43%)	3(6)	3(6)	16(31)
Temporal lobe	8(16%)	0	3(6)	5(10)
Frontal lobe	5(10%)	1(2)	1(2)	3(6)
Parietal lobe	6(12%)	0	3(6)	3(6)
Occipital lobe	2(4%)	0	0	2(4)
Insula	2(4%)			
Cerebellar hemisphere	2(4%)			
Laminar terminalis	4(8%)			
Suprasellar	4(8%)			
Anterior falx and others	6(12%)			

**Table 3:** Association of cerebral infarcts with leptomeningeal enhancement, vasculitis, vasospasm and outcome.

	Cerebral infarcts(n = 34)	No cerebral infarct(n = 17)	p value
Vasculitis (n, %)			
Yes	13 (45%)	2 (18%)	0.16
No	16 (55%)	9 (82%)	
Vasospasm (n, %)			
Yes	6 (21%)	0 (0%)	0.16
No	23 (79%)	11 (100%)	
Leptomeningeal enhancement (n, %)			
Yes	29 (85%)	7 (41%)	
No	5 (15%)	10 (59%)	0.002
Functional outcome at 3 months (n, %)			
Poor outcome (MRS 3–6)	27 (79%)	9 (53%)	
Good outcome (MRS 0–2)	7(21%)	8 (47%)	0.10
